# *Cordyceps militaris* Induces Immunogenic Cell Death and Enhances Antitumor Immunogenic Response in Breast Cancer

**DOI:** 10.1155/2020/9053274

**Published:** 2020-09-03

**Authors:** Xingguo Quan, Beom Seok Kwak, Ji-Young Lee, Jin Hee Park, Anbok Lee, Tae Hyun Kim, SaeGwang Park

**Affiliations:** ^1^Department of Microbiology and Immunology, College of Medicine, Inje University, Busan 47392, Republic of Korea; ^2^Department of Surgery, Ilsan Hospital, College of Medicine, Dongguk University, Ilsan 10326, Republic of Korea; ^3^Department of Internal Medicine, College of Medicine, Inje University, Busan 47392, Republic of Korea; ^4^Department of Surgery, College of Medicine, Inje University, Busan 47392, Republic of Korea

## Abstract

*Cordyceps militaris* has been widely used as a traditional medicine in East Asia. Its effects against breast cancer have been reported previously. However, whether *C. militaris*-induced breast cancer cell death is immunogenic remains unelucidated. This study aimed to determine whether ethanolic extracts of *C. militaris* (CM-EE) could induce immunogenic cell death (ICD) in breast cancer immunotherapy to improve the efficacy of immune checkpoint inhibitors. Human and mouse breast cancer cells were treated with various concentrations of CM-EE for 72 h, and cytotoxicity was measured using the sulforhodamine B assay. Flow cytometry was used to assess cell death with annexin V/7-AAD staining and measure the surface exposure of damage-associated molecular pattern (DAMP) molecules including calreticulin, HSP70, and HSP90. Western blot for cleaved poly (ADP-ribose) polymerase (PARP) was used to confirm apoptotic cell death. The immunogenicity of CM-EE-induced dead cells was evaluated using the CFSE dilution assay. CM-EE reduced the viability of human (MCF7, MDA-MB-231, HS578T, and SKBR3) and mouse (4T1-neu-HA, TUBO-HA, and TUBO-P2J-HA) breast cancer cells. The IC_50_ was 25–50 *µ*g/ml in human breast cancer cells and 10–50 *µ*g/ml in mouse breast cancer cells at 72 h. CM-EE-treated breast cancer cells were positively stained by annexin V, cleaved PARP, and cleaved caspase 3/7 which were increased upon CM-EE treatment. Surface exposure of DAMP molecules was increased in dose- and time-dependent manners. The CFSE dilution assay revealed that dendritic cells fed with CM-EE-treated breast cancer cells successfully stimulated tumor-specific T cell proliferation without inhibiting DC function and T cell proliferation. The expression of PD-L1 mRNA and protein level was increased in dose-dependent manners. In addition, CM-EE also potentiated the cytotoxic activity of tumor-specific T cells. CM-EE can induce immunogenic and apoptotic cell death in breast cancer cells, and it is a good candidate for cancer immunotherapy and may improve the efficacy of immune checkpoint inhibitors.

## 1. Introduction

Immune checkpoint inhibitors (ICIs), such as programmed cell death 1 (PD-1)/programmed cell death ligand 1 (PD-L1) and cytotoxic T-lymphocyte antigen-4 (CTLA-4) inhibitors, have shown promising clinical outcomes over the past several years. It exhibits antitumor activity by inhibiting tumor cells from evading immunosurveillance and reinvigorating antitumor immune response [1]. The FDA has approved ICIs for various malignancies such as melanoma, non-small-cell lung cancer, renal cell carcinoma, Hodgkin's lymphoma, head and neck cancer, and breast cancer. The overall response rate of ICIs alone in for each malignancy does not exceed approximately 30% [2]. There is difference among the various subtypes of malignancies, and the response rate of ICIs alone for advanced breast cancer is low [3–5].

Several efforts have been made to overcome the low response rate of ICIs alone in patients with breast cancer. Using combinations of modalities such as chemotherapy, radiotherapy, targeted monoclonal antibodies, and endocrine therapy is a promising strategy to increase the efficacy of ICIs [6]. Immunogenic cell death (ICD) induced by combinations of these modalities may facilitate treatment with ICIs. Tumor cells exposed to ICD inducers undergo apoptosis; simultaneously, damage-associated molecular patterns (DAMPs) such as calreticulin (CRT), adenosine triphosphate (ATP), high mobility group box 1 (HMGB1), and heat shock protein (HSP)70 and 90 are exposed or released on the cell surface [7, 8]. These DAMPs activate dendritic cells (DCs) and stimulate the presentation of tumor antigens to T cells [9, 10], thereby inhibiting tumor cell growth.

Organic medicines are now being used in antitumor treatment owing to their strong cytotoxicity and low side effects [11]. *Cordyceps militaris* is a fungus found in the caterpillar stage of moths and has widely been used as a traditional medicine in East Asia [12–14]. *C. militaris* has a strong pharmacological activity and has received extensive attention; it exerts immunomodulatory, anti-inflammatory, antimicrobial, and antitumor effects [15–18]. It increases the expression of major histocompatibility complex (MHC) and MHC-restricted antigen presentation in dendritic cells and leads to increases in T cell proliferation [19].

If *C. militaris* can induce ICD, it may be a potential combination partner for improving the efficacy of ICIs in breast cancer. Therefore, in this study, we investigated if *C. militaris* can induce ICD in human and mouse breast cancer cells.

## 2. Materials and Methods

### 2.1. Materials

Ethanol extract of *C. militaris* (CM-EE) was provided by DONG-A ST (Seoul, Korea). EE was prepared by incubating fresh fruiting bodies or mycelia of *C. militaris* in 50% ethanol at 37°C for 3 days. The extracts were filtered, concentrated, sterilized, and dried as previously described [20].

### 2.2. Cell Culture

Human (MDA-MB-231, MCF-7, HS578T, and SKBR-3) and mouse (4T1-neu-HA, TUBO-HA, and TUBO-P2J-HA) breast cancer cells were cultured in Dulbecco's modified Eagle medium (DMEM) or RPMI 1640 medium supplemented with 10% fetal bovine serum (FBS), 100 U/ml penicillin, and 100 *µ*g/ml streptomycin under a humidified atmosphere containing 5% CO_2_ at 37°C and passaged every 2 days.

### 2.3. Cytotoxicity Assay

Cytotoxicity of CM-EE on human and mouse breast cancer cells was measured with using an in vitro toxicology assay kit that was sulforhodamine B-based (SRB assay, Sigma-Aldrich, USA). Briefly, cells were seeded in 96-well flat-bottom plates at a density of 1–10 × 10³ cells/well and treated with various concentrations of CM-EE (0–200 *µ*g/ml) for 72 h and then subjected to the SRB assay. Absorbance was measured using a multimode microplate reader (SpectraMax, USA) at 565 nm.

### 2.4. APC-Annexin V and 7-AAD Assay

Cell death type (apoptosis or necrosis) was analyzed using the APC-Annexin V apoptosis detection kit from BioLegend (San Diego, CA, USA). Briefly, the cells were seeded in 6-well plates at a density of 1–10 × 10^4^ cells/well and incubated at 37°C overnight. The cells were then treated with various concentrations of CM-EE (0–200 *µ*g/ml) for 72 h and stained with APC-Annexin V and 7-aminoactinomycin D (7-AAD), after which the cells were incubated for 15 min at room temperature away from light and analyzed using the FACS CantoII flow cytometer (BD, USA).

### 2.5. PARP and Caspase 3/7 Western Blot Analysis

Total protein was isolated from the cells (human breast cancer cell lines: MDA-MB-231 and MCF-7) using radioimmunoprecipitation analysis (RIPA) lysis buffer (Thermo Scientific, USA). The isolated protein was separated by SDS-PAGE and transferred onto nitrocellulose (NC) membranes. The membranes were blocked with 5% nonfat dry milk in TBST (a mixture of tris-buffered saline and Tween 20) and incubated with cleaved PARP (Poly ADP-ribose polymerase) antibody (Cell Signaling, USA), PARP antibody (Cell Signaling, USA), caspase 3/7 antibody (Cell Signaling, USA), and HRP-conjugated anti-rabbit IgG (Cell signaling, USA). The signal was then detected using a bioimaging analyzer system (Amersham Imager 600, GE Healthcare, USA). The membranes were stripped and probed with GADPH antibody as a loading control.

### 2.6. Flowcytometry Analysis

The cell surface exposures of CRT, HSP70, HSP90, and human PD-L1 proteins were detected using flowcytometry. Briefly, the cells were treated with various concentrations of CM-EE (0–200 *µ*g/ml) for 72 h and stained with primary antibody for CRT (Cell Signaling, USA), HSP70, or HSP90 (Abcam, United Kingdom) and secondary antibody (Alexa flour 750-conjugated anti-mouse Fc antibody, Abcam, United Kingdom). For PD-L1 expression, cells were treated with CM-EE for 48 h and stained with APC-conjugated anti-PD-L1 antibody (ebioscience, USA). Fluorescence was analyzed using the FACS CantoII flow cytometer and FlowJo software (BD, USA).

### 2.7. RT-PCR Assay

Total RNA was extracted using TRIzol reagent (Life Technologies, Carlsbad, CA, SUA) according to manufacturer's protocol. The yield and the quality RNA were analyzed using a NanoDrop 2000 Spectrophotometer (Thermo Science, USA). Extracted RNA was subjected complementary cDNA synthesis using TOPscript RT DryMIX synthesis kit (Enzynomics, Korea) according to the manufacturer's instructions and using Veriti 96-Well Thermal Cycler (Applied Biosystems, USA). For DNA amplification Accupower PCR Premix (BIONEER, Korea) was used according to the manufacturer's instructions. The primer PD-L1 was used (forward; 5′-TTGGGAAATGGAGGATAAGA-3′, reverse; 5′-GGATGTGCCAGAGGTAGTTCT-3′), GAPDH (forward; 5′-GGAGCGAGATCCCTCCAAAAT-3′, reverse; 5′-GGCTGTTGTCATACTTCTCATGG-3′) for reaction mix, then samples were loaded on agarose gel without adding a loading-dye mixture, and electrophoresis was performed. The gel was then detected using a bioimaging analyzer system (Amersham Imager 600, GE Healthcare, USA).

### 2.8. Generation of Bone Marrow-Derived DCs

Bone marrow cells were isolated from the femurs and tibias of 5-6-week-old female Balb/c mice. The cells were resuspended in RPMI 1640 supplemented with 10% heat-inactivated FBS, 100 U/ml penicillin, 100 *µ*g/ml streptomycin, and immature bone marrow-derived DCs were differentiated with recombinant GM-CSF (10 ng/ml, R&D SYSTEMS, USA) in a humidified 5% CO_2_ incubator at 37°C. On days 3, 5, 7, and 9 of culture, floating cells were gently removed, and fresh, warmed medium with mouse recombinant GM-CSF (10 ng/ml) was added. On day 10, nonadherent cells were harvested and used for in vitro experiments.

### 2.9. T Cell Proliferation and CTL Activity Assay

T cell proliferation was analyzed using the CFSE dilution assay. Splenocytes were isolated from CL4-HA TCR transgenic mice, and the cells were labelled with CFSE for 4 min at 37°C. CFSE-labeled splenocytes were cultured in the presence of BMDC fed with CM-EE-treated cancer cells for 72 h. For activation, 0.1 *µ*g/ml HA_512–520_ peptide (IYSTVASSL) was used. The cells were harvested and analyzed using the FACS CantoII flow cytometer and FlowJo software. Cytotoxic T cell (CTL) killing function was analyzed using the CFSE and 7-AAD staining. Briefly, the splenocytes of CL4 mouse were seeded in 96-well round-bottom plates at a density of 2 × 10^6^ cells/well and for activation treated with 0.2 *µ*g/ml HA-peptide, incubated at 37°C for 72 h. The mouse breast cancer cells (4T1-neu-HA, TUBO-HA cells) were labelled 1*µ*M CFSE, co-cultured with activated T cells, and also treated with various concentrations of CM-EE (0–200 *µ*g/ml) for 8 h and stained with 7-AAD, after which the cells were incubated for 15 min at room temperature away from light and analyzed using the FACS CantoII flow cytometer (BD, USA).

### 2.10. Statistical Analysis

Between-group differences were analyzed using unpaired *t* test. Error bars represent ± standard deviation. Data were analyzed using GraphPad Prism (Version 6 for Windows; GraphPad Software; San Diego, CA). Unless specified, *p* < 0.05, 0.10, and 0.001 have been noted with ^*∗*^, ^*∗∗*^, and ^*∗∗∗*^, respectively. Differences that are not statistically significant have been left unnoted.

## 3. Results

### 3.1. CM-EE Has Cytotoxic Effects on Human and Mouse Breast Cancer Cells

**Figure 1:**
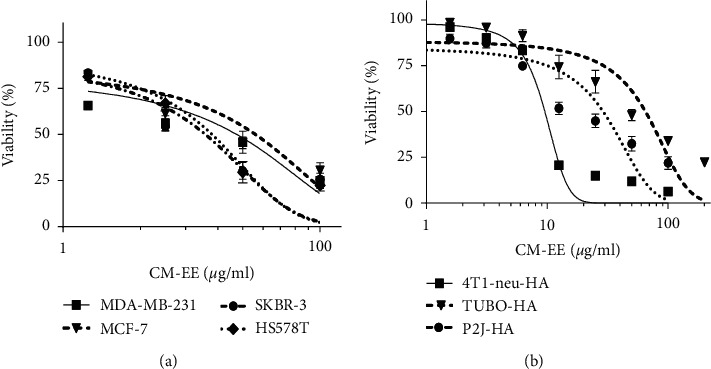
Cell cytotoxicity of CM-EE on human and mouse breast cancer cell lines. (a) Human breast cancer cell lines (MDA-MB-231, SKBR-3, HS578T, and MCF-7) and (b) mouse breast cancer cell lines (P2J-HA, 4T1-neu-HA, and TUBO-HA) were treated with various concentrations (0–200 *μ*g/ml) of CM-EE for 72 h, and cell viability was determined using the SRB assay. The results are presented as mean ± standard deviation (SD) for three independent experiments. CM-EE : *Cordyceps militaris* ethanolic extract.

To investigate the cytotoxic effects of CM-EE on human and mouse breast cancer cell lines, the cells were treated with various concentrations of CM-EE (0, 0.78125, 1.5625, 3.125, 6.25, 12.5, 25, 50, 100, and 200 *µ*g/ml; cell viability was evaluated using the SRB assay (Figure 1). CM-EE reduced the viability of the entire tested human (Figure 1(a)) and mouse (Figure 1(b)) breast cancer cell lines in a dose-dependent manner. The IC_50_ (half maximal inhibitory concentration) of CM-EE was 55.96 ± 4.62 *µ*g/ml for MCF-7, 35.76 ± 3.01 *µ*g/ml for SKBR-3, 37.91 ± 2.71 *µ*g/ml for HS578T, and 47.33 ± 5.00 *µ*g/ml for MDA-MB-231 in human and 9.55 ± 0.24 *µ*g/ml for 4T1-neu-HA, 28.92 ± 2.12 *µ*g/ml for TUBO-P2J-HA, and 64.78 ± 4.40 *µ*g/ml TUBO-HA in mouse breast cancer cells.

### 3.2. CM-EE Induced Apoptotic Cell Death in Human and Mouse Breast Cancer Cells

**Figure 2:**
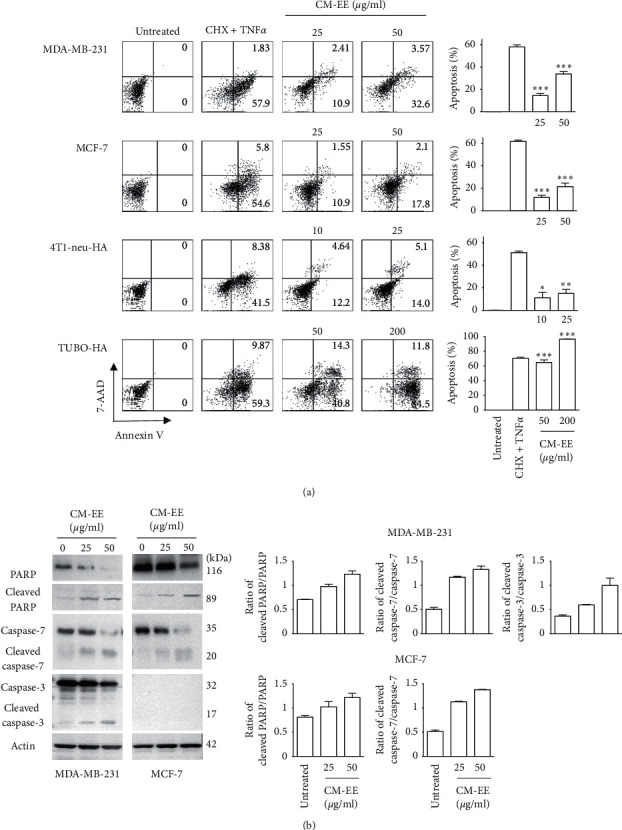
CM-EE induced apoptotic cell death in human and mouse breast cancer cells. The cells were treated with CM-EE or CHX (cycloheximide: 2 ng/ml) + TNF*α* (tumor necrosis factor-*α*: 10 *µ*g/ml) for 72 h and then stained with APC-Annexin V and 7-AAD (a) or western blotting was conducted with PARP, caspase 3/7 antibodies (b). (a) Representative dot plot for Annexin V and 7-AAD staining and apoptotic cell death of human and mouse breast cancer cells. The results are presented as mean ± standard deviation (SD) for three independent experiments. Unpaired *t* test was used for statistical analysis. ^*∗*^*p* < 0.05, ^*∗∗*^*p* < 0.01, and ^*∗∗∗*^*p* < 0.001 compared with control. (b) Western blot image and analysis of protein levels for total and cleaved PARP, caspase 3/7.

To investigate whether the cell death type induced by CM-EE is apoptosis or necrosis, Annexin V and 7-AAD staining were conducted. The cells were treated with different concentrations (MDA-MB-231 and MCF-7: 25 or 50 *µ*g/ml, 4T1-neu-HA: 10 or 25 *µ*g/ml, and TUBO-HA: 50 or 200 *µ*g/ml) of CM-EE for 72 h and then stained with APC-Annexin V and 7-AAD. The stained cells were analyzed by flow cytometry.  Figure 2(a) shows representative dot blots for Annexin V/7-AAD staining. 7-AAD single-stained cells were not detected, and most stained cells were Annexin V single or Annexin V and 7-AAD double stained. These data suggest that CME induced apoptotic cell death in human and mouse breast cancer cells. In addition, Annexin V-positive cells increased with increasing doses of CM-EE. To confirm that CM-EE induces apoptotic cell death, the level of PARP and caspase 3/7 protein was measured using western blotting (Figure 2(b)). In both MCF7 and MDA-MB-231 cells, the levels of cleaved PARP protein were increased in a dose-dependent manner. In MDA-MB-231 cells, the level of cleaved caspase 3/7 protein was increased in a dose-dependent manner.

### 3.3. CM-EE Induced the Surface Exposure of Calreticulin and Heat Shock Protein 70 or 90 in Human and Mouse Breast Cancer Cells

**Figure 3:**
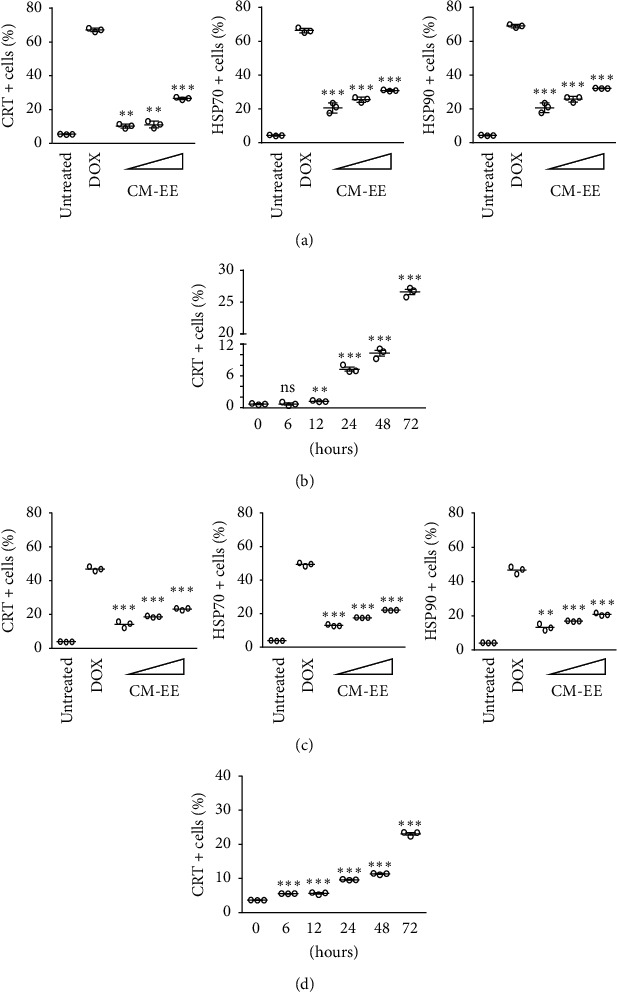
CM-EE elicits the apoptotic cell surface exposure of CRT, HSP70, and HSP90 on human and mouse breast cancer cells. (a) Human breast cancer cells (MDA-MB-231 cells) and (c) mouse breast cancer cells (4T1-neu-HA cells) were treated with dose-dependent concentrations of CM-EE (0–100 *µ*g/ml) for 72 h and then stained with anti-CRT, anti-HSP70, and anti-HSP90 and analyzed by flow cytometry. (b) MDA-MB-231 cells and (d) 4T1-neu-HA cells were treated with 100 *µ*g/ml CM-EE for 1, 3, 6, 12, 24, 48, and 72 h and then stained with anti-CRT and analyzed by flow cytometry. The results are presented as mean ± standard deviation (SD) for triple replicates. The unpaired *t* test was used for statistical analysis. ^*∗*^*p* < 0.05, ^*∗∗*^*p* < 0.01, and ^*∗∗∗*^*p* < 0.001 compared with control.

To test whether CME-induced cell death could be ICD, cell surface exposures of the ICD markers calreticulin and HSP70 or HSP90 were evaluated using flow cytometry (Figure 3). By treatment with doxorubicin, a strong ICD inducer, MDA-MB-231 cells showed a positive rate of >60% of CRT and HSP70 or HSP90 surface exposed cells compared with untreated controls. The abovementioned CM-EE-treated cells also showed that, as the drug-dependent concentration gradually increased, the surface exposure of CRT, HSP70, and HSP90 also gradually increased; however, the number of positive cells was less than that of the doxorubicin-treated cells (Figure 3(a)). Up to 12 h after drug treatment, the surface exposure of the CRT did not increase significantly compared with that of the untreated control; however, the surface exposure of the CRT gradually increased from 24 h to 72 h (Figure 3(b)). Although there were fewer CRT- and HSP70 or HSP90-positive cells in the CM-EE treatment group than in the doxorubicin treatment group, the possibility that CM-EE could induce ICD in breast cancer cells remained. The surface exposure of CRT and HSP70 or HSP90 was also treated by CM-EE in mouse breast cancer cells (4T1-neu-HA cells), as in the treatment of human breast cancer cells, which increased as the drug-dependent concentration gradually increased (Figure 3(c)). Over time, the surface exposure of the CRT was similar to that in human breast cells (Figure 3(d)). In other human breast cells (MCF-7 cells and TUBO-HA cells), we also found that, after CM treatment, the surface exposure of CRT, HSP70, and HSP90 gradually increased as the drug concentration increased compared with the untreated control (Supplementary Figures 1(a) and 1(b)). Therefore, human and mouse breast cancer cells treated with CM-EE were exposed to ICD markers on the cell surface.

### 3.4. CM-EE-Induced Apoptotic Cells Increase T Cell Proliferation in Mouse Breast Cancer Cells

**Figure 4:**
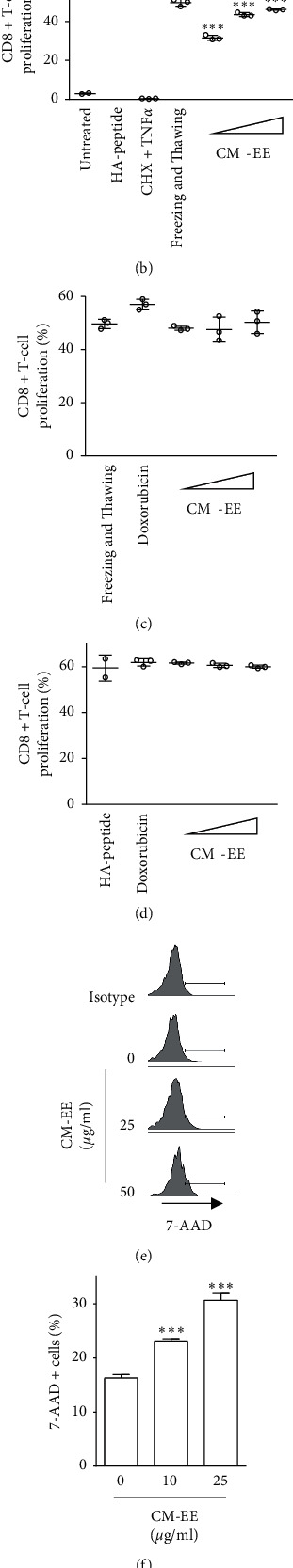
CFSE T cell proliferation assay ((a)–(d)) and CTL activity assay ((e)–(g)). (a) Schematic illustration of T cell proliferation assay to evaluate the effects of CM-EE on immunogenic cell death, DC cross-presentation, and T cell proliferation ((b)–(d)). (b) HA expressing mouse breast cancer cells (4T1-neu-HA) were treated with CM-EE (0–50 *µ*g/ml) for 72 h. Apoptotic and silent death control was prepared with CHX (2 ng/ml) + TNF*α* (10 *µ*g/ml) for 72 h. Necrotic and immunogenic cell death control was prepared by repeating three times the freezing and thawing cycle (FT). Pretreated 4T1-neu-HA cells were fed to BMDCs for overnight. Ag-fed BMDCs were co-cultured with CFSE-labelled CL4 splenocytes for 72 h (c) Bone marrow-derived DCs were fed with FT-4T1-neu-HA cells and co-cultured with CFSE-labelled CL4 splenocytes for 72 h with or without CM-EE (10–50 *μ*g/ml) or doxorubicin (0.5 *μ*M). (d) CFSE-labelled CL4 splenocytes were stimulated with HA-peptide (0.2 *μ*g/ml) with or without CM-EE (10–50 *μ*g/ml). T cell proliferation was evaluated using flow cytometry. ((e)–(g)) CL4 splenocytes were activated with HA-peptide (0.2 *μ*g/ml) for 72 h and co-cultured with CFSE-labelled 4T1-neu-HA or TUBO-HA cells for 8 h. Dead tumor cells stained with 7-AAD and analyzed with flowcytometry. (e) Representative histograms for 7-AAD staining of CFSE-positive cells. ((f)-(g)) Percentage of dead 4T1-neu-HA (f) and TUBO-HA (g) cells by activated HA-specific CL4 splenocytes. Data are presented as mean ± standard deviation (SD) for three independent experiments. The results are presented as mean ± standard deviation (SD) for triple replicates. The unpaired *t* test was used for statistical analysis. ^*∗∗∗*^*p* < 0.001 compared with control. DCs: dendritic cells.

The above experimental data indicate the possibility that CM-EE induces ICD in breast cancer cells; however, this aspect remains unclear. To confirm that CM-EE can induce ICD, a hemagglutinin (HA) antigen-specific CD8^+^ T cell proliferation system was recruited (Figure 4(a)). DCs fed with CM-EE-treated 4T1-neu-HA cells induced the proliferation of CL4 mouse T cells in a dose-dependent manner (*p* < 0.001) (Figure 4(b)). We also obtained similar results for TUBO-HA cells (Supplementary Figure 2). These data provide concrete evidence that CM-EE induces ICD.

### 3.5. CM-EE Does Not Inhibit the T Cell Activation Function of Dendritic Cells and the Proliferating Potential of T Cells

It is clear that CM-EE induced ICD in human and mouse breast cancer cells. However, the ICD inducing potential of CM-EE is ineffective when CM-EE inhibits DC and T cell functions. To evaluate whether CM-EE can inhibit DCs, CM-EE treatment was applied when DCs were fed with necrotic cells or when T cells were co-cultured with DCs fed with necrotic cells. DCs fed with necrotic cells successfully stimulated the proliferation of CL4 CD8^+^ T cells, and the potential of DCs to stimulate T cell proliferation was not changed by treatment with CM-EE of up to 100 *µ*g/ml (Figure 4(c)). By stimulation with HA-peptide, CL4 CD8^+^ T cells showed approximately 60% of T cell proliferation, and the proliferation rate was not changed by CME addition of up to 100 *µ*g/ml (Figure 4(d)). These results revealed that CME does not inhibit the functions of DCs in T cell stimulation and the proliferating potential of T cells.

### 3.6. CM-EE Increased the CTL Activity of Tumor-Specific T Cells

To evaluate whether CM-EE can effect on cytotoxic activity of tumor-specific T cells, we studied CTL activity assay with/without CM-EE. Unlike DC cross-presentation and T cell proliferation, CM-EE increased the CTL activity of tumor-specific CL4 splenocytes dose-dependently (Figures 4(e)–4(g)). These results revealed that CM-EE can help antitumor response of T cells.

### 3.7. CM-EE Increased the Expression of PD-L1 mRNA and Protein in MDA-MB-231 Cell

**Figure 5:**
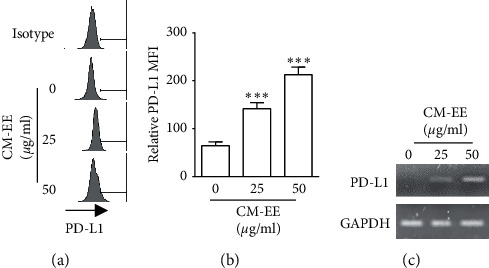
CM-EE increased the expression of PD-L1 mRNA and protein in MDA-MB-231 cell. MDA-MB-231 cells were treated with CM-EE (0–50 *µ*g/ml) for 48 h and then the expression of PD-L1 protein ((a)-(b)) and mRNA (c) were evaluated using flowcytometry or RT-PCR. (a) Representative histograms for PD-L1 expression on MDA-MB-231 cells. (b) Fluorescence intensity of PD-L1 expression. The results are presented as mean ± standard deviation (SD) for triple replicates. The unpaired *t* test was used for statistical analysis. ^*∗∗∗*^*p* < 0.001 compared with control. (c) Images of agarose gel electrophoresis of PD-L1 mRNA RT-PCR.

In order to clarify the relationship between CM-EE and immune checkpoint, we determined that CM-EE can induce the expression of PD-L1 molecules. By the treatment of CM-EE for 48 h, MDA-MB-231 cells showed increased surface expression of PD-L1 molecules (Figures 5(a) and 5(b)). RT-PCR experiments revealed that CM-EE treatment increased the expression of PD-L1 mRNA (Figure 5(c)). These results revealed that CM-EE can synergize with PD-L1 blockade.

## 4. Discussion

In this study, we investigated whether CM-EE can induce ICD in human and mouse breast cancer cells. With the advancement of science, more and more cell death mechanisms have been discovered, the most representative of those being apoptotic and necrotic cell death. Some cell death mechanisms similar to necrosis, such as necroptosis, have recently been discovered [21]. As already well known, apoptotic cell death is programmed cell death and is characterized by the participation of apoptotic proteins that maintain membrane integrity in vitro [22]. In this experiment, we used tumor necrosis factor alpha (TNF*α*) and cycloheximide (CHX) to induce apoptosis; however, we found that these did not increase T cell proliferation (Figure 4(b) and Supplementary Figure 2). Necrotic cell death is nonprogrammed cell death and is characterized by membrane rupture and inflammation. Cell integrity is destroyed in necrotic cell death, and the death is passive [23]. We prepared necrotic cells after freezing and thawing at high temperatures in our experiments. Necrosis increased the proliferation of T cells (Figure 4(b) and Supplementary Figure 2). However, in our experiments, we induced human and mouse breast cancer cell death after treatment with CM-EE. The type of cell death detected by Annexin V APC fluorescence was apoptosis (Figure 2(a)); apoptotic marker cleaved PARP, cleaved caspase-3, and cleaved caspase-7 which were gradually increased in human breast cancer cells (Figure 2(b)), and it gradually increased the T cell proliferation in a drug-dependent manner (Figure 4(b) and Supplementary Figure 2). This is indicative of another kind of cell death, similar to necrosis, which is necroptosis. Necroptosis is a programmed form of necrosis, or inflammatory cell death, so it possesses the characteristics of both apoptotic and necrotic cell death. In particular, the nuclear material released during the process of necroptosis seems to be more immunogenic than the apoptotic material [24]. This explains our experimental results well, so we cannot rule out the fact that CM-EE induces necroptosis.

CM-EE-induced ICD could promote the release of DAMP molecules and the proliferation of CD 8^+^ T cells, resulting in enhanced tumor immunogenicity. In clinical practice, there are numerous ongoing researches on combination modalities to improve the response of ICIs. Among those, radiation, chemotherapeutic agents such as anthracyclines, and microtubule-stabilizing agents like ICD inducers show favorable outcomes when combined with ICIs [25]. Indeed, atezolizumab/nab-paclitaxel combinations and pembrolizumab/eribulin combinations showed significant clinical benefits in patients with metastatic breast cancer [26, 27]. We found CM-EE increased the expression of PD-L1 mRNA and protein levels (Figure 5). Surprisingly, we also found CM-EE increased CTL activity of tumor-specific T cells (Figures 4(e)–4(g)), even though it is not clear that CM-EE makes tumor cells more susceptible to CTLs or help T cells in killing tumor cells.

Our data showed that the exposure of DAMP molecules and CD8^+^ T cell proliferation is less increased by CM-EE than by doxorubicin. Unfortunately, it seems that the efficacy of CM-EE for ICD induction is lower than that of doxorubicin, a traditional ICD inducer. However, doxorubicin results in adverse events including nausea/vomiting, alopecia, cardiotoxicity, and liver injury [28], and some patients cannot use doxorubicin owing to its side effects. *C. militaris* has no significant adverse events in healthy people, though this is only a small-scale study and further investigation about its toxicity is warranted [29]. We speculate that CM-EE might be a useful substitute in patients who are contraindicated to doxorubicin. Many compounds such as cordycepin, mannitol, ergosterol, and polysaccharides are isolated from *C. militaris* [15, 20]. Actually, cordycepin is well-known as the anticancer effects on various cancers, however, it is not clear that cordycepin-induced cancer cell death is immunogenic or not. Further study to evaluate which component induces ICD is required.

## 5. Conclusions

Taken together, CM-EE inhibits the growth of human and mouse breast cancer cells, induces ICD, potentiates CTL activity, and increases expression of PD-L1. As the concentration- and time-dependence of CM-EE increase, CRT, HSP70, and HSP90 exposed to the cell surface also gradually increase and this is a significant feature of ICD. We also proved that CME-induced cell death is immunogenic via an in vitro cross-presentation experiment. Our experimental data indicate that CM-EE is a good candidate for cancer immunotherapy and can improve the efficacy of ICIs.

## Data Availability

This is an open access article distributed under the Creative Commons Attribution License, which permits unrestricted use, distribution, and reproduction in any medium, provided the original work is properly cited.

## Supplementary Materials

Supplementary Materials Supplementary Figure 1. CM-EE elicits apoptotic cell's surface exposure of CRT, HSP70, and HSP90 on human and mouse breast cancer cells. (a) Human breast cancer cells (MCF-7 cells) and (b) mouse breast cancer cells (TUBO-HA cells) were treated with indicated doses of CM-EE (0–200 *µ*g/ml) for 72 h and stained with anti-CRT, anti-HSP70, and anti-HSP90 and analyzed by flow cytometry. The results are presented as mean ± standard deviation (SD) for triple replicates. ^*∗*^*p* < 0.05, ^*∗∗*^*p* < 0.01, and ^*∗∗∗*^*p* < 0.001 compared with control. Supplementary Figure 2: CM-EE treated mouse breast cancer cells can enhance the cross-presentation of dendritic cells. CL4 CD8+ T cell proliferation stimulated by dendritic cells fed with CM-EE treated on TUBO-HA cells. The results are presented as mean ± standard deviation (SD) for triple replicates. ^*∗∗∗*^*p* < 0.001 compared with control.
