# Supplementary figures and images for: Cordyceps militaris Induces Immunogenic Cell Death and Enhances Antitumor Immunogenic Response in Breast Cancer
Source: Evid Based Complement Alternat Med. 2020 Sep 3;2020:9053274. doi: 10.1155/2020/9053274 (PMC7486645; doi:10.1155/2020/9053274)

**a**

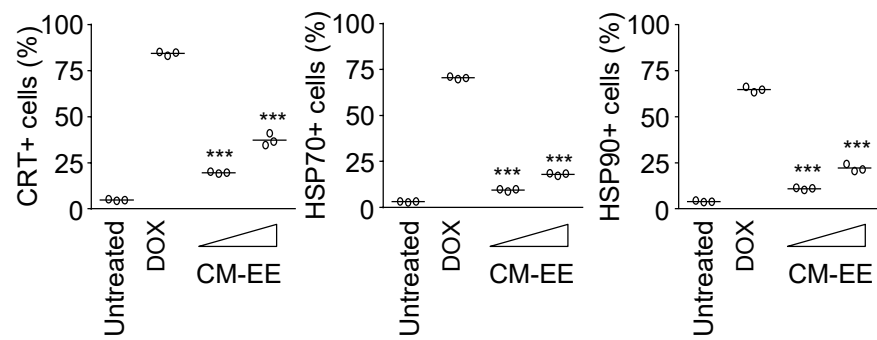

**b**

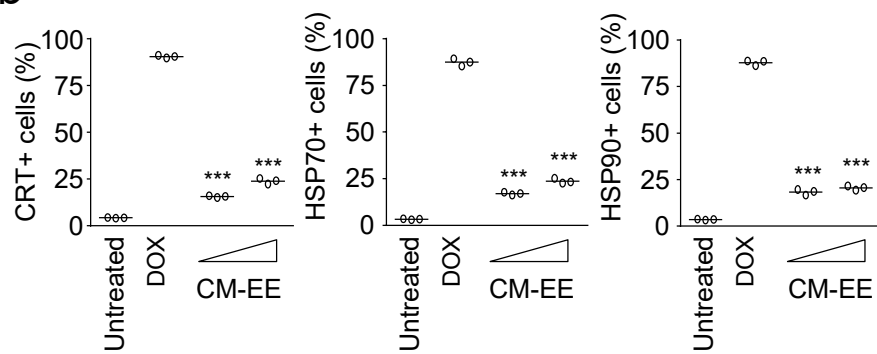

Supplementary Figure 1

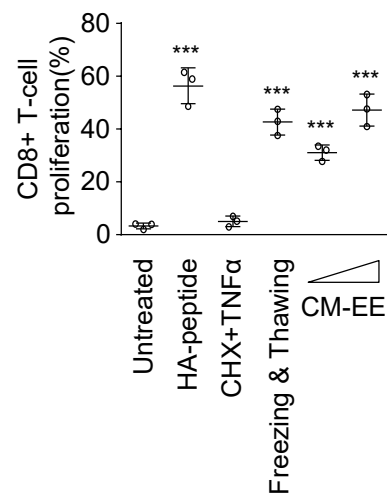

Supplementary Figure 2

Supplement: Supplementary Materials — Supplementary Figure 1. CM-EE elicits apoptotic cell's surface exposure of CRT, HSP70, and HSP90 on human and mouse breast cancer cells. (a) Human breast cancer cells (MCF-7 cells) and (b) mouse breast cancer cells (TUBO-HA cells) were treated with indicated doses of CM-EE (0–200 µg/ml) for 72 h and stained with anti-CRT, anti-HSP70, and anti-HSP90 and analyzed by flow cytometry. The results are presented as mean ± standard deviation (SD) for triple replicates. ∗p < 0.05, ∗∗p < 0.01, and ∗∗∗p < 0.001 compared with control. Supplementary Figure 2: CM-EE treated mouse breast cancer cells can enhance the cross-presentation of dendritic cells. CL4 CD8+ T cell proliferation stimulated by dendritic cells fed with CM-EE treated on TUBO-HA cells. The results are presented as mean ± standard deviation (SD) for triple replicates. ∗∗∗p < 0.001 compared with control. [file 9053274.f1.pdf]
